# Crack-Width Threshold-Guided Design of Self-Healing Polyethylene Fiber-Reinforced Engineered Cementitious Composites (ECCs)

**DOI:** 10.3390/polym18192345

**Published:** 2026-09-25

**Authors:** Zhigang Zhang, Xiangwen Lei, Jamal A. Abdalla, Rami A. Hawileh, Qiang Shen, Yuanchuan Chen

**Affiliations:** 1 School of Civil Engineering, Chongqing University, Chongqing 400045, China; 2 State Key Laboratory of Safety and Resilience of Civil Engineering in Mountain Area, Chongqing University, Chongqing 400045, China; 3 Department of Civil Engineering, American University of Sharjah, Sharjah P.O. Box 26666, United Arab Emirates; jabdalla@aus.edu (J.A.A.);; 4 School of Urban Construction Engineering, Chongqing University of Arts and Sciences, Chongqing 402160, China

**Keywords:** polyethylene fiber, engineered cementitious composites (ECC), self-healing, exposure regimes, mechanical properties

## Abstract

Polyethylene (PE) fibers enable engineered cementitious composites (ECCs) to achieve tensile strain-hardening and multiple-cracking behavior through fiber bridging; however, the relatively large crack widths that can develop in PE fiber-reinforced high-strength ECC (HS-ECC) may limit its autogenous self-healing capability. This study therefore establishes a direct link between PE fiber-mediated crack control, matrix micromechanical tailoring, and autogenous self-healing. The critical crack-width thresholds for reliable self-healing were first determined using water sorptivity tests combined with wet–dry cyclic exposure. The results showed that transport properties could be restored to nearly their original levels when crack widths were below approximately 50 μm in tap water and 60 μm in seawater. Based on these thresholds, waste fly ash ceramsite (FAC) was subsequently incorporated to tailor the matrix fracture characteristics and promote saturated multiple cracking under PE fiber bridging. Replacing 20% of quartz sand with FAC increased the tensile strain capacity from 2.75% to 7.14% and the crack number from 17 to 61, while reducing the average crack width from 110 μm to approximately 49 μm. The refined crack pattern enabled reliable autogenous self-healing under wet–dry cycling, with seawater exhibiting particularly favorable healing behavior. These findings provide a micromechanics-based strategy for designing sustainable PE fiber-reinforced HS-ECC with enhanced ductility, crack control, and intrinsic self-healing capability.

## 1. Introduction

Polyethylene (PE) fibers have been increasingly employed as high-performance polymer reinforcement in cementitious composites because of their high tensile strength, elastic modulus, chemical stability, and corrosion resistance [1,2,3]. In particular, PE fibers play a critical role in the development of engineered cementitious composites (ECC), also referred to as strain-hardening cementitious composites (SHCC), which exhibit high tensile ductility and multiple-cracking behavior under tensile loading [4,5,6,7,8]. Unlike conventional fiber-reinforced concrete, ECC is designed based on micromechanical principles through the coordinated tailoring of the polymer fibers, cementitious matrix, and fiber–matrix interface [9]. During loading, PE fibers bridge developing matrix cracks and transfer tensile stress across the crack surfaces, while interfacial debonding and fiber sliding allow additional cracks to form rather than localizing deformation into a single dominant crack [10]. This polymer fiber-bridging mechanism enables ECC to achieve tensile strain-hardening behavior and maintain cracks at the microscale [11].

The ability of polymer fibers to regulate crack formation is particularly important for the durability of cementitious composites. Cracks provide preferential pathways for water, chloride ions, and other aggressive species to penetrate the cementitious matrix, accelerating deterioration of concrete infrastructure [2,3]. Meanwhile, sufficiently narrow cracks can undergo autogenous self-healing through continued hydration of unhydrated cementitious particles, pozzolanic reactions, and precipitation of healing products such as calcium carbonate [10,12]. Consequently, the tight crack-width control provided by polymer fibers creates favorable conditions for ECC to exploit its intrinsic self-healing capability, potentially reducing transport properties after cracking and extending the service life of structures [11].

Self-healing of cementitious materials can generally be classified into autonomous and autogenous approaches [13]. Autonomous self-healing relies on externally introduced healing components, such as microcapsules, vascular systems, or bacteria. Upon cracking, healing agents can be released or activated to fill the crack and restore material properties [14,15,16,17]. Although these approaches can provide considerable healing efficiency, challenges remain regarding the long-term viability of bacteria, premature rupture of capsules during mixing, compatibility between healing agents and the cementitious matrix, and the probability that propagating cracks intersect the incorporated healing components [13,18]. In contrast, autogenous self-healing requires no additional healing agents and utilizes the intrinsic hydration and precipitation processes of the cementitious matrix. This approach is therefore particularly attractive for polymer fiber-reinforced ECC, in which the formation of numerous fine cracks provides favorable conditions for naturally occurring healing reactions.

Despite its potential, autogenous self-healing is strongly dependent on crack width because the amount of available healing products is inherently limited. A fundamental issue is therefore the maximum crack width that can be reliably healed. Considerable discrepancies exist in the literature regarding this critical threshold. Yang et al. [19] reported that cracks up to approximately 50 μm could be completely healed, as indicated by recovery of permeability to the level of uncracked specimens, and similar observations were subsequently reported by Kan et al. [20] and Guan et al. [21]. Lepech et al. [22] demonstrated autogenous healing of cracks of approximately 60 μm. In contrast, Lahmann et al. [23], based on experimental observations and numerical simulations, suggested that cracks as wide as 113 μm could exhibit autogenous self-healing. These discrepancies may originate from differences in healing criteria, exposure conditions, mixture compositions, initial crack characteristics, and evaluation methods. Therefore, the critical crack-width threshold for reliable autogenous self-healing remains insufficiently defined.

Clarifying this threshold is particularly important for PE fiber-reinforced high-strength ECC (HS-ECC). In conventional ECC, polyvinyl alcohol (PVA) fibers are commonly used because their hydrophilic surfaces develop relatively strong interactions with the cementitious matrix, contributing to tight crack-width control, with typical crack widths often below 60 μm [24]. PE fibers, in contrast, are hydrophobic and exhibit relatively weak chemical affinity with the cementitious matrix. Although the high tensile strength and elastic modulus of PE fibers enable the development of HS-ECC with excellent tensile ductility, the combined effects of the PE fiber–matrix interface and the stronger, tougher matrix of HS-ECC can result in crack widths approaching approximately 100 μm [25,26,27,28]. If cracks of this magnitude can undergo reliable autogenous self-healing, as suggested by Lahmann et al. [23], conventional PE-HS-ECC may already possess considerable intrinsic healing potential. However, if reliable healing is restricted to approximately 50–60 μm, further micromechanical tailoring of the PE fiber-reinforced composite is required to refine its crack pattern. Establishing the critical healing threshold is therefore essential for linking polymer fiber-induced crack control with the autogenous self-healing performance of HS-ECC.

The exposure environment introduces another important uncertainty. Most previous investigations of autogenous self-healing have been conducted using tap water or laboratory water-curing conditions, whereas PE fiber-reinforced ECC has considerable potential for marine infrastructure because both PE fibers and the cementitious matrix offer favorable resistance to aggressive environments. Seawater contains various dissolved ions that may alter the precipitation, composition, and morphology of healing products and consequently affect both healing efficiency and the critical healable crack width [12,14]. However, the relationship between crack width and autogenous self-healing of PE-HS-ECC under seawater exposure remains insufficiently understood. Establishing environment-dependent crack-width thresholds is therefore important for designing PE fiber-reinforced ECC for marine applications.

Once the critical healable crack width is identified, the next challenge is to tailor the PE fiber-reinforced composite so that its crack widths consistently remain below this threshold. According to the micromechanical design principles of ECC, multiple cracking and crack-width control are governed not only by fiber properties and fiber–matrix interactions but also by the fracture characteristics and flaw distribution of the matrix. In this context, lightweight fly ash ceramsite (FAC), produced from fly ash waste, provides a potential means of modifying matrix fracture behavior while simultaneously improving material sustainability. The introduction of FAC may reduce matrix fracture toughness and modify the flaw-size distribution, thereby activating a larger number of matrix flaws during tensile loading. Such changes are expected to promote saturated multiple cracking, distribute tensile deformation over more cracks, and consequently reduce individual crack widths. This provides a potential strategy for tailoring the crack pattern of PE-HS-ECC toward the requirements for reliable autogenous self-healing.

Accordingly, this study aims to establish a direct link between PE fiber-mediated crack control, matrix micromechanical tailoring, and autogenous self-healing in HS-ECC. Water sorptivity tests combined with wet–dry cyclic exposure were first employed to quantify the recovery of transport properties and determine the critical crack-width thresholds for autogenous self-healing in tap water and seawater. Based on the identified thresholds and the micromechanical design principles of ECC, waste FAC was subsequently incorporated to tailor the matrix fracture characteristics and refine the crack pattern of PE fiber-reinforced HS-ECC. The resulting changes in tensile behavior, crack distribution, and self-healing performance were evaluated, while the healing products formed under different exposure environments were characterized using energy-dispersive X-ray spectroscopy (EDS). Finally, the mechanisms through which FAC-induced matrix modification influences PE fiber-mediated multiple cracking and autogenous self-healing were elucidated. The findings provide a micromechanics-based strategy for designing sustainable PE fiber-reinforced cementitious composites with enhanced ductility, crack control, and intrinsic self-healing capability.

## 2. Experimental Programs

### 2.1. Raw Materials

In this study, the primary components of the ECC mixture included CEM-45 Portland cement, fly ash, silica sand (SS), silica fume (SF), fly ash ceramic (FAC), superplasticizer (SP), water (W), and polyethylene (PE) fibers. FA was obtained from the Huaneng Luohuang Power Plant (Chongqing, China), and SF was purchased from Weilin Te Industrial Co., Ltd. (Quanzhou, China). Cement was supplied by the Chongqing Xiaonanhai Cement Plant (Chongqing, China). The bulk densities of FAC and SS were 0.8 and 1.6 g/cm^3^, respectively. The PE fibers had a density of 0.97 g/cm^3^, a nominal tensile strength of 3000 MPa, and an elastic modulus of 120 GPa. The diameter and length of the PE fibers were 26 μm and 12 mm, respectively. The particle size distribution of solid ingredients is shown in Figure 1. The chemical compositions of the main cementitious materials are presented in Table 1.

Table 2 lists the mix proportion of ECC mixtures. The binder materials consisted of 51 wt.% Portland cement, 41 wt.% fly ash, and 8 wt.% silica fume. In the reference ECC mixture (Ref.-ECC), the sand-to-binder ratio was maintained at 0.3. In the another mixture (FAC-ECC), 20% of the silica sand was volumetrically replaced with fly ash ceramics (FAC) particles, which were introduced to serve as artificial flaws aimed at narrowing crack widths. The 20% FAC replacement was adopted as a representative design case to examine the feasibility of crack-width refinement rather than to determine an optimum FAC dosage. The water-to-binder ratio was fixed at 0.2. The dosage of polyethylene (PE) fibers was maintained at 2% by volume.

### 2.2. Mixing Procedure

To prepare the ECC mixtures, all solid ingredients were first placed into a mixer and dry-mixed for 2 min. Subsequently, a pre-mixed solution of water and superplasticizer (SP) was slowly poured into the mixer and blended for 8 min until homogeneous slurry was obtained. At this stage, the mixer speed was reduced to a low setting, and the polyethylene (PE) fibers were gradually added. The speed was then adjusted to a medium level, and mixing continued for an additional 5 min to ensure uniform fiber dispersion. The resulting mixture was then cast into molds that had been lightly coated with a release agent. After 24 h, the specimens were demolded and transferred to a laboratory for curing. The curing was performed at a temperature of 25 ± 3 °C and a relative humidity of 65 ± 10%, both of which were monitored and recorded using a temperature-humidity data logger.

### 2.3. Testing Procedures

In this study, uniaxial tensile tests were conducted on three dumbbell-shaped specimens for each mixture in accordance with the JSCE recommendations [29]. The tests were performed under displacement control using a universal testing machine (WDW-20H, Jinan Chuanbai Instrument Equipment Co., Ltd., Jinan, China) with a load capacity of 20 kN. Two external linear variable differential transformers (LVDTs) were mounted on opposite sides of the specimen to monitor deformation within the gauge region. The loading rate was maintained at 0.5 mm/min. Loading was continued beyond the peak tensile stress until a clear tensile-softening response was observed, after which the test was terminated. The tensile strain was calculated from the average displacement measured by the two LVDTs divided by the gauge length. After testing, the crack pattern within the gauge region was recorded, and the widths of representative cracks were measured using an optical microscope with a resolution of 1 μm.

The water sorptivity test measures the rate of water ingress into unsaturated concrete, expressed as the change in mass per unit contact area over time, and is widely regarded as a standard method for evaluating capillary suction-related water transport [4]. This rate of ingress is significantly influenced by the presence of cracks, as they provide more direct and convenient pathways for water to penetrate the material. Therefore, evaluating the variation in sorptivity is considered an appropriate and effective means to characterize the influence of crack self-healing behavior on transport properties.

Prior to the test, the ECC beams were dried in a vacuum oven (DZF-6020BZ, Shanghai Yixin Scientific Instrument Co., Ltd., Shanghai, China; rated power: 1100 W) at 60 °C for three days until a constant mass was achieved. This procedure was used to establish a comparable initial moisture condition and minimize the influence of internal moisture on the test results. Although identical preconditioning was applied to all specimens, drying at 60 °C may induce minor drying-related microstructural changes. Therefore, the healing results reported herein should be interpreted within the adopted conditioning protocol. Subsequently, all beams underwent pre-cracking through a four-point bending test, as illustrated in Figure 2c,d. The pre-cracked beams were then cut to obtain water sorptivity test specimens in the form of cubes (50 × 50 × 50 mm) containing visible cracks. Additionally, two uncracked specimens were prepared to serve as control samples for comparison.

Following the four-point bending test, the cracked ECC cubes were subjected to the water sorptivity test by partially immersing their bottom surfaces (3–5 mm) in water. Notably, all lateral surfaces of the specimens were sealed with epoxy resin, and the top surface was covered with a plastic sheet, leaving only the bottom surface unsealed, as illustrated in Figure 2d. During the test, the mass of each specimen was recorded at specified time intervals of 1, 5, 10, 20, 30, 60, 120, 180, 240, and 360 min, in accordance with ASTM C1585 [30]. Based on the measured mass changes, the water sorptivity index (*I*) was calculated using Equation (1), where W_s_ corresponds to the slope of the linear regression line fitting the cumulative water absorption versus the square root of time.

(1) $$Ι=\frac{m_{a}-m_{0}}{A\rho_{w}}$$

where I denotes the water absorption (mm), m_0_ refers to the initial mass of sample (g), m_a_ refers to the mass of sample after water sorptivity test (g). A is the area in contact with water (mm^2^), *ρ_w_* denotes the density of water (g/mm^3^).

After the dog-bone specimens were subjected to tensile loading, the test was stopped once tensile softening was observed after the peak load. The damaged specimens were then subjected to wet–dry curing in either tap water or seawater to simulate healing conditions under tidal environments. During this process, an industrial microscope with an accuracy of 1 μm was employed to monitor the self-healing behavior of cracks with varying widths under different numbers of wet–dry cycles (0, 1, 7, and 14 cycles). Each cycle consisted of 24 h of water curing followed by 24 h of air curing.

Artificial seawater was prepared in accordance with ASTM D1141-98, and its chemical composition is listed in Table 3. Deionized water (9 L) was used as the base solution, in which 245 g of sodium chloride and 40.1 g of anhydrous sodium sulfate were dissolved under continuous stirring until complete dissolution was achieved. Subsequently, 200 mL of stock solution No. 1 was slowly introduced and thoroughly mixed, followed by the addition of 100 mL of stock solution No. 2 with continued stirring. The solution was then diluted to a final volume of 10 L, and the pH was adjusted to 8.2 using 0.1 N sodium hydroxide solution to ensure chemical stability and accuracy.

## 3. Results and Discussion

### 3.1. Water Sorptivity Test Result

Figure 3 illustrates the temporal variation in water absorption for specimens containing 0 to 5 cracks. The uncracked specimen cured in tap water is designated as T0, while T1 denotes the specimen with a single crack under the same curing condition, with the numbering convention continuing accordingly. Similarly, specimens with two and three cracks cured in seawater are labeled S2 and S3, respectively. The corresponding water sorptivity index (W_s_) values for all specimens are summarized in Table 4.

As shown in Figure 3, the presence of cracking generally increased water absorption compared with the uncracked specimens. However, the sorptivity did not vary strictly with crack number, indicating that crack number alone is insufficient to fully characterize the effect of cracking on water transport. Other geometrical characteristics, including crack width, crack length, connectivity, and total crack opening, may also contribute to the measured sorptivity. In the test series, the cracked specimens were classified according to the number of visible cracks. Individual crack widths and lengths were not systematically recorded prior to the sorptivity tests; therefore, the respective contributions of crack number and crack geometry cannot be quantitatively separated. The crack number should thus be regarded as a qualitative descriptor of the cracking condition rather than an independent governing parameter. Accordingly, the sorptivity results are primarily used in this study to characterize the recovery of water-transport resistance after wet–dry healing, rather than to establish a quantitative crack-number–sorptivity relationship.

As summarized in Table 4, prior to self-healing curing, the water sorptivity index (Ws) was increased from 0.00029 (T0) to 0.00041 (T1) and 0.00235 (T5), corresponding to increases of approximately 41% and 710%, respectively. In other cases, the Ws values for T2, T3, and T4 were 0.00155, 0.00147, and 0.00222, respectively. A similar trend was also observed in specimens subjected to seawater curing. These results clearly indicate that the presence of cracks significantly against the resistance to capillary suction-driven water absorption in concrete. Such elevated absorption can adversely affect the durability of concrete structures, since water ingress is widely recognized as a primary contributor to long-term deterioration in infrastructure [31].

Encouragingly, compared to the non-cured specimens, the Ws values of the pre-cracked samples were significantly reduced after undergoing wet–dry cycle curing, regardless of whether they were cured in seawater or tap water. Specifically, the Ws value of samples T1–T5 decreased from an initial range of 0.00041–0.00235 to 0.00021–0.00096, approaching that of the un-cracked specimen T0 (0.00029). This can be attributed to the formation of autogenous self-healing products, as demonstrated in Figure 4, which reduced or blocked the pathways for water ingress. Furthermore, a more pronounced reduction in *W_s_* was observed in seawater-cured specimens, for instance, after self-healing conditioning, the *W_s_* value of specimen S5 was minimized to 0.00187 from 0.004013 prior to self-healing, representing 53.4% decrement, suggesting a potentially enhanced autogenous self-healing capacity in marine environments.

Figure 5 illustrates the differences in the water sorptivity index, denoted as Dws. This index represents the difference between the Ws values of pre-cracked and uncracked specimens, denoting the contribution of Ws value gaining from cracks in specimens. Specifically, Dws-a and Dws-b refer to the values after and before self-healing, respectively. As shown in Figure 5, a clear reduction in the crack-induced water sorptivity was observed after wet–dry healing under both tap-water and seawater exposure. This result demonstrates that autogenous healing effectively reduced the water-transport pathways associated with cracking. However, the initial sorptivity values of the tap-water and seawater groups were different, and the initial crack geometries of the two groups were not strictly matched. Therefore, the absolute Ws values and their reductions were not used for a direct quantitative comparison of healing efficiency between the two exposure environments. Instead, the sorptivity results are interpreted primarily as evidence of water-transport recovery within each specimen group.

Overall, the above findings indicate that the presence of cracks is a cause for concern, as an increase in crack number facilitates the ingress of water and aggressive ions. Encouragingly, even in the presence of such cracks, wet–dry cycle curing was found to mitigate water ingress by promoting crack sealing through self-healing processes. This recovery was observed under both tap-water and seawater exposure conditions. Because the initial crack geometries were not strictly equivalent between the two groups, the relative influence of the exposure environment cannot be isolated from the sorptivity results alone and is therefore further evaluated through crack-width-resolved observations in Section 3.2.

### 3.2. Self-Healing Phenomenon Observations

As previously discussed, water absorption in cracked specimens can be effectively reduced due to the occurrence of self-healing. As outlined in Section 1, the self-healing of cracks is highly dependent on crack width. It is generally accepted that narrower cracks are more likely to promote self-healing. This phenomenon can be reasonably explained by the fact that self-healing products are primarily generated from further hydration of unreacted cementitious materials, which are not sufficient to seal wide cracks. Accordingly, a threshold crack width is expected to exist beyond which effective self-healing cannot occur.

In this section, four representative crack widths (approximately 15 μm, 40 μm, 50 μm, and 90 μm) were selected to investigate the self-healing behavior of ECC under different curing environments (tap water and seawater). The aim was to gain deeper insight into the influence of crack width on the self-healing performance of ECC and to identify the critical crack width required for effective autogenous healing under varying environmental conditions.

Figure 6 shows the progression of crack self-healing under wet–dry cycles in tap water for cracks of various widths. After 1 wet–dry cycle, no significant formation of healing products was observed for cracks ranging from 10 to 90 μm. However, after 7 cycles, noticeable differences in healing behavior were evident. Cracks with widths of 10.9 μm and 40.5 μm appeared to be fully sealed by white self-healing products along the crack path. In contrast, no such sealing was observed in the cracks measuring 58.1 μm and 85.5 μm. The white material progressively filling the cracks is attributed to newly formed autogenous healing products, likely associated with carbonate precipitation and continued hydration. These products gradually occupy the available crack space and contribute to the observed reduction in crack opening. Their detailed composition is further discussed in Section 3.3.2.

It is worth noting that although small amounts of white healing products were locally observed within the 58.1 μm crack, this healing behavior may have been influenced by the presence of fibers. Fibers are known to act as nucleation sites that facilitate cement hydration and the formation of calcium carbonate. It was observed that localized self-healing occurred specifically at points where fibers were embedded within the crack, consistent with the crack locations captured in the specimen images prior to the wet–dry cycles (i.e., at 0 cycles).

After 14 wet–dry cycles, further self-healing was observed in cracks narrower than 50 μm, with crack traces becoming less visible and healing products even overflowing from the crack openings. Encouragingly, self-healing behavior was also observed in the 58.1 μm crack; however, minor crack traces still remained unfilled. In contrast, for the 85.5 μm crack, visible crack traces were retained, indicating limited or no healing.

By comparing the self-healing behavior across different crack widths, it is evident that ECC possesses self-healing capabilities. However, crack width plays a crucial role in determining the effectiveness of this behavior. For instance, a critical transition in self-healing performance was observed around a crack width of 58 μm, beyond which complete closure becomes difficult to achieve. From these observations, it can be inferred that cracks narrower than approximately 40 μm exhibit significant healing, whereas residual cracks remain visible in the case of 58.1 μm, suggesting that cracks within this range may not be fully sealed. Closer examination of the 58.1 μm crack revealed that its lower portion had undergone complete healing after 14 wet–dry cycles. Measurements showed that the healed region had a width of approximately 52.2 μm. The results show that, within the crack-width range investigated in this study, effective autogenous healing was observed for cracks up to approximately 50 μm in tap water, which aligns well with the results reported by Yang and Kan [19,20].

Figure 7 displays the self-healing process in cracks under wet–dry cycles in seawater. Remarkably, after only one cycle, cracks narrower than 50 μm were found to be rapidly sealed by self-healing products. This early-stage sealing is expected to effectively block the ingress of water and chloride ions, thereby preventing the internal accumulation of corrosive agents commonly associated with durability deterioration. Such prompt healing behavior is particularly attractive for enhancing the long-term durability of concrete marine structures.

In the case of the 58.6 μm crack, partial sealing by white self-healing products was observed; however, visible crack traces still remained. For the 98 μm crack, the presence of unsealed crack openings was even more apparent under visual inspection, indicating a limited self-healing response at wider crack widths.

With the increase in wet–dry cycles, the accumulation of self-healing products within the cracks became more evident after 7 cycles. However, the cracks measuring 58.6 μm and 98 μm remained incompletely healed. After 14 cycles, slight changes were observed across the different crack widths. Specifically, the 58.6 μm crack was found to be fully healed, whereas the 98 μm crack still exhibited a prominent opening that had not been sealed by the white self-healing products.

These observations further indicate that crack closure is a progressive process rather than an instantaneous event. For initially wider cracks, partial precipitation during the early healing stages reduces the residual crack opening, which may create more favorable conditions for subsequent filling during later wet–dry cycles. This behavior was particularly evident for the 58.6 μm crack under seawater exposure, which remained incompletely healed after 7 cycles but was completely sealed after 14 cycles. In contrast, the approximately 98 μm crack remained visibly open even after 14 cycles. These results suggest that extended exposure may promote further closure of residual defects; however, whether initially wider cracks can ultimately achieve complete closure cannot be established from the present 14-cycle observation period.

A comparison of the crack self-healing behavior under seawater and tap water curing conditions reveals notable differences. As shown in Figure 6 and Figure 7, the number of wet–dry cycles required for complete crack sealing varies significantly between the two environments. For cracks smaller than 50 μm, full healing was achieved in both cases; however, seawater curing proved to be more effective, as complete crack closure was observed after just 1 wet–dry cycle. In contrast, specimens cured in tap water required up to seven cycles to achieve similar results.

Moreover, it was observed that the seawater curing environment exhibited a slightly more relaxed threshold for crack width compared to tap water. Specifically, the 58 μm crack was fully sealed by self-healing products under seawater wet–dry cycles, while under tap water curing, the same crack remained visibly open. These findings highlight the enhanced self-healing performance of ECC in marine-like environments. The present observations indicate qualitatively different healing kinetics. Under tap-water exposure, little visible healing occurred during the first cycle, followed by substantial closure of narrow cracks by seven cycles. Under seawater exposure, cracks below approximately 50 μm exhibited much faster initial closure. Because only four discrete observation times were available, however, a quantitative healing-rate law was not derived.

Therefore, it can be concluded that, compared to tap water curing, the self-healing behavior of ECC cracks is more favorably promoted under seawater exposure. Within the crack-width range investigated in this study, the threshold crack width for effective autogenous healing can be extended from about 50 μm to 60 μm. The identification of this critical value is of substantial importance for foundational design across varying environmental conditions. With a clearly defined crack width threshold for self-healing, it becomes feasible to employ micromechanical design principles in the development of ECC mixtures tailored to meet this criterion. This, in turn, contributes to improving the long-term durability of infrastructure and reducing the frequency of maintenance interventions.

It should be emphasized that the above values represent approximate healing boundaries inferred from the crack widths examined in the present study rather than statistically exact universal thresholds. A larger number of independently controlled crack widths would be required to establish a probabilistic critical crack width.

### 3.3. HS-ECC Mixture Design for Realizing Self-Healing

As emphasized in Section 1, the crack width in PE fiber-reinforced HS-ECC is typically limited to approximately 100 μm. It is demonstrated in Section 3.2 that autogenous self-healing is seldom achieved under these conditions. Accordingly, restricting the development of crack in width is essential, as excessive cracking can impair the self-healing effect of ECC. In this section, for realizing the effective self-healing, the HS-ECC mixture with narrow crack in width (<50 μm) was re-designed by incorporating fly ash ceramics (FAC) to replace 20% of the silica sand, in this way, the FAC likely act as “artificial flaws” in ECC, which reduces the fracture toughness of ECC matrix, allowing the initiation of crack at a relatively lower tensile stress corresponded to narrower crack opening, as a result, the width of cracks in ECC specimen under tension can be minimized.

#### 3.3.1. Tensile Properties

Figure 8 presents the uniaxial tensile stress–strain curves of two batches of ECC mixtures prepared in this study. The curves exhibit the expected pseudo-strain hardening behavior. Specifically, after the formation of the first crack and the associated initial stress drop, the ECC able to maintain a stable load-carrying capacity. As the tensile load increased further, additional cracks were progressively initiated. The repeated stress drops and recoveries observed in the curves (seen in Figure 8) provide direct evidence of successive crack formation. This desirable behavior can be attributed to the fiber-bridging effect arising from the interaction between the fibers and surrounding matrix. The beneficial role of fiber bridging in restricting crack propagation and improving crack control and toughness has also been reported in other fiber-reinforced cementitious systems [32]. The maximum fiber-bridging stress in specimen exceeds the matrix cracking strength, thereby enabling multiple cracking rather than localized failure. This behavior is sustained until the fiber bridging effect is exhausted and surpassed by the strength of the crackable cross-section, beyond which new crack initiation is suppressed, thereby leading to tensile softening. Figure 9 summarizes the first cracking strength (i.e., the stress corresponding to the initiation of the first crack), the peak tensile strength, and the associated tensile strain capacity.

From Figure 9, the Ref.-ECC mixture can be classified as a high-ductility concrete, as it achieved a tensile strain capacity of 2.75%, which is approximately 300 times that of conventional concrete. However, this performance remains unsatisfactory, as the strain capacity is significantly lower than that of other PE-ECC reported in the literature [25,26,33]. This deficiency may be attributed to the insufficient redundancy in satisfying the micromechanical design criteria (i.e., the strength criteria) [34], likely due to the relatively high matrix cracking strength. The development of saturated multiple cracking behavior is inhibited by these properties, which delay crack initiation and propagation, as will be further discussed later in this paper. More critically, the crack width observed in the Ref.-ECC mixture was approximately 110 μm. As discussed in Section 3.2, this width exceeds the threshold, typically considered favorable for promoting self-healing behavior. Consequently, the durability of structures utilizing this mixture may be significantly compromised.

Accordingly, there is a pressing need to enhance the tensile ductility of PE-ECC while simultaneously reducing crack width, thereby enabling the material to fully leverage its intrinsic self-healing capabilities.

As revealed in Figure 9, the incorporation of FAC sensibly increased the strain capacity of PE-ECC. Specifically, admixing FAC improved the 28-day strain capacity of PE-ECC by 160%, which reached 7.14%. The likely mechanism is as follows: the FAC acting as artificial flaw in ECC mixture, which may have contributed to a reduction in matrix cracking strength. Under conditions where the fiber bridging stress remains nearly constant, as illustrated in Figure 9, this reduction plays a favorable role in meeting the design criteria for saturated multiple cracking. The tensile strength of ECC is primarily governed by the fiber bridging capacity [1], and in this case, it experienced only a minor reduction of approximately 7%, decreasing from 7.88 MPa in Ref.-ECC to 7.42 MPa in FAC-ECC. This reduction is relatively lower compared to the decrease in matrix cracking strength, which dropped by approximately 22% from 5.82 MPa to 4.53 MPa.

The combined effect of a relatively stable fiber bridging capacity and a notably reduced matrix cracking strength led to an increase in the redundancy of the strength criterion. According to micromechanics based ECC design principles, this redundancy facilitates the formation of multiple cracks [34]. A greater level of redundancy allows more sections within the composite to reach the cracking stage before the ECC attains its peak strength. This contributes to improved ductility and toughness in the concrete structure.

As for the crack pattern, it is generally accepted that a greater number of cracks indicates a more desirable tensile cracking behavior in ECC dumbbell specimens for attaining high ductility. On the other hand, as discussed in Section 3.2, crack width is a critical parameter that directly influences the self-healing performance of ECC. Table 5 provides a summary of the crack characteristics of representative ECC specimens. Figure 10 shows the crack width distribution of representative dumbbell specimens, in which the red curves represent the corresponding Weibull fitting functions. All the above crack data were measured after unloading at the peak tensile load.

As shown in Figure 10 and Table 5, the incorporation of FAC significantly increased the number of cracks initiated in the ECC specimens. Compared to the 17 cracks observed in representative Ref.-ECC specimen, the number increased to 61 when FAC was added, representing a 259% rise. This enhancement in multiple cracking behavior is consistent with the proposed role of FAC as additional crack-initiation sites within the matrix, effectively promoting the formation of distributed cracks, as illustrated in Figure 8.

Additionally, the formation of narrower cracks was found to be promoted by the incorporation of FAC. The average crack width in Ref.-ECC was measured to be 110 μm, which, according to the findings in Section 3.2, is unfavorable for the development of self-healing behavior. Notably, the average crack width in the FAC-ECC specimen was effectively narrowed to 49 μm, bringing it much closer to the critical threshold required for crack self-healing. This result clearly demonstrates the successful realization of the original design objective.

With regard to the underlying mechanism, the narrowing of crack width can be reasonably explained based on the micromechanical design principles of ECC [34]. Figure 11 illustrates a representative fiber bridging stress-crack opening (*σ*-*δ*) curve. Symbol 1 represents the original values, whereas symbol 2 indicates the variation in crack width. The incorporation of FAC as artificial flaws is believed to reduce the matrix cracking strength, as previously discussed in Figure 9. As observed in the *σ*-*δ* curve, the crack width was narrowed in response to the reduction in matrix stress. Importantly, the peak bridging stress was assumed to remain unchanged, given that the addition of FAC had minimal impact on the overall tensile strength (seen in Figure 9). This mechanism has also been reported by other researchers and is consistent with previous findings in the field [35]. It should be emphasized that the fiber-bridging curve, matrix fracture toughness, and fiber–matrix interfacial properties were not directly measured; Figure 11 therefore represents a conceptual interpretation rather than direct experimental evidence.

#### 3.3.2. Self-Healing Behavior

As outlined in Section 3.3.1, the incorporation of FAC effectively narrowed the crack width in PE-ECC. In this section, dumbbell specimens of FAC-ECC were subjected to 14 cycles of wet–dry curing to investigate the self-healing behavior. Figure 12 presents the morphology of cracks within the gauge region of the dumbbell specimens after curing in either tap water or seawater.

As shown in Figure 12a, all cracks on the dumbbell specimen, except for the primary failure crack due to its significant opening, were sealed. This is evidenced by the appearance of white filamentous structures, which represent the formation of self-healing products. This self-healing behavior clearly validated the previously stated finding that cracks with a width of approximately 50 μm are capable of being effectively healed. The presence of unhealed wide cracks can be attributed to localized damage that developed after the specimen reached its peak tensile stress, during which continued loading promoted further crack propagation.

For specimens subjected to seawater curing, the self-healing phenomenon was observed to be significantly more pronounced compared to those cured in tap water. As shown in Figure 12b, white self-healing products were observed to nearly overflow from the crack surfaces, indicating a highly notable healing effect. This result is consistent with the findings presented in Section 3.2, where it was demonstrated that seawater curing conditions promote more favorable self-healing behavior. It should be noted that these specimens were loaded through the tensile test and therefore contained both distributed strain-hardening cracks and a localized post-peak failure crack. Consequently, this experiment was intended as a qualitative validation of the crack-width-guided mixture-design concept rather than a direct simulation of service-level pre-cracking. Future work should employ controlled unloading at prescribed tensile strain levels to evaluate self-healing under representative service crack conditions.

SEM and EDS analyses were conducted to further examine the self-healing products formed within cracks under different environments. In Figure 13a (tap water), the yellow dashed lines indicate the original top surface of the crack. It can be observed that, at different crack widths, the self-healing products mainly consisted of irregular crystals, including plate-like and prismatic forms, by which the cracks were almost completely sealed. The EDS results revealed that these self-healing products were primarily composed of Ca, Si, O, and C, as shown in Figure 14 and Table 6, with Ca/Si ratios ranging from 2 to 4. This finding suggests that the major products were likely CaCO_3_ and C-S-H phases [36]. However, definitive phase identification would require complementary characterization such as X-ray Diffraction, Raman spectroscopy, or Fourier Transform Infrared Spectroscopy.

Figure 13b presents the morphology of crack healing after seawater curing. Unlike the complex morphology observed in tap water, the self-healing products in seawater exhibited nearly identical appearances. EDS analysis indicated that the products were primarily composed of C, Ca, and O, demonstrating that they mainly consisted of calcium carbonate. As shown in Figure 14, the Ca/Si ratio was far from 4, which confirmed that the products were predominantly calcium carbonate [36]. The mechanism underlying the formation of these self-healing products will be discussed in the following section.

### 3.4. Self-Healing Behavior Mechanism

Figure 15 illustrates the self-healing mechanism of cracks in specimens subjected to different curing environments (tap water and seawater). As depicted in Figure 15a, unhydrated cement particles within the matrix continue to hydrate over time. Simultaneously, carbon dioxide from the atmosphere dissolves into the water, forming carbonate ions. These ions react with calcium ions present in the matrix to generate calcium carbonate, while the hydration process produces calcium silicate hydrate (C-S-H) gel. Together, the cracks are sealed by these self-healing products.

In addition, autogenous healing is also governed by mass transport and reaction kinetics. Under the relatively stagnant immersion conditions adopted herein, the transfer of atmospheric CO_2_ into the aqueous phase may be more limited than that under well-mixed conditions. However, the alternating wet–dry cycles repeatedly expose the crack surfaces to both water and air. During the wet period, continued hydration, dissolution, and ion transport can occur, whereas drying promotes evaporation and local concentration of dissolved species, creating favorable conditions for subsequent precipitation within the crack. Repetition of these processes may progressively contribute to crack filling.

In terms of crack width, narrower cracks exhibit a higher likelihood of being completely healed. This is primarily due to the reduced volume of hydration products required to fill narrow cracks, which in turn shortens the time needed for effective healing. It should be noted that excessively wide cracks are less likely to be fully sealed, as the amount of unhydrated cement available in the matrix is limited. This explains why, as shown in Figure 6, narrower cracks require less time to heal and are more likely to be completely closed, whereas wider cracks tend to remain partially open.

In the case of seawater curing, in addition to the self-healing products observed under tap water conditions, the abundance of ions in seawater also serves as a supplementary source of reactants for the self-healing process. For instance, the presence of carbonate (CO_3_^2−^), sulfate (SO_4_^2−^), and calcium (Ca^2+^) ions in seawater may promote the formation of self-healing products such as ettringite and calcium carbonate. This explains why, as discussed in Section 3.2, a wider crack threshold for self-healing was observed under seawater curing compared to that under tap water curing. Moreover, since these essential ions are readily available in the seawater environment, the time required for crack closure can be substantially reduced. This is because the healing reaction no longer relies solely on the dissolution of atmospheric carbon dioxide or the leaching of calcium ions from the cement matrix. As shown in Figure 6 and Figure 7, this mechanism allows for a more efficient and accelerated self-healing process in seawater. Solution chemistry, particularly pH, may further influence the healing kinetics. The artificial seawater was initially adjusted to pH 8.2; however, pH evolution during the subsequent wet–dry cycles was not monitored. Contact with the cementitious matrix may alter the local alkalinity through dissolution and leaching of hydration products, thereby affecting carbonate speciation and the precipitation of Ca-bearing products.

Future studies should therefore combine time-resolved measurements of pH, Ca^2+^ concentration, dissolved inorganic carbon, and other relevant ionic species with quantitative monitoring of crack geometry to establish the relationship between reaction kinetics and macroscopic crack closure. These considerations also suggest that self-healing ECC may be further developed through a coupled design strategy combining mechanical crack-width control with chemical tailoring of the matrix. Maintaining sufficient reactive phases and a favorable local chemical environment may enhance autogenous healing, although such modifications must be balanced against matrix strength, fracture behavior, and PE fiber–matrix interactions to preserve strain-hardening performance.

## 4. Conclusions

In this study, the effect of crack self-healing behavior on water absorption was investigated. To determine the critical crack-width threshold that enables autogenous self-healing in PE-ECC, wet–dry cycle test was conducted on specimens with varying crack widths under different curing environments. Furthermore, a PE-ECC mixture featuring narrow crack widths, suitable for self-healing, was successfully developed. The main conclusions are summarized as follows:

(1) Compared to uncracked specimens, the water sorptivity index was significantly increased with the number of cracks. Fortunately, this negative effect was largely mitigated by the autogenous self-healing behavior activated during wet–dry cycles, which restored the water absorption performance to a level comparable to that of the uncracked specimens.
(2) Under tap water curing, cracks narrower than approximately 50 μm were fully sealed by self-healing products after 7 cycles, whereas wider cracks remained visibly open even after 14 cycles, indicating incomplete healing. In contrast, seawater curing significantly accelerated the self-healing process: cracks narrower than 50 μm were completely sealed after 1 cycle, and the threshold crack width for effective self-healing increased to 60 μm, with cracks of this width fully closed after 7 cycles.
(3) A low-crack-width PE-ECC was successfully developed through the incorporation of FAC, resulting in a 160% increase in tensile strain capacity to 7.14%, with negligible effect on tensile strength. The average crack width was reduced from 110 μm in the reference PE-ECC mixture to 49 μm in the FAC-modified ECC mixture. These narrowed cracks were fully healed under wet–dry cycling. EDS analysis indicated that the self-healing products formed under seawater curing were primarily composed of CaCO_3_, whereas those formed under tap water curing consisted of a combination of C-S-H and CaCO_3_.

Beyond the specific experimental results, this study demonstrates that autogenous self-healing of PE-ECC can be approached as a coupled mechanical–chemical design problem. From the mechanical perspective, the matrix should be tailored to promote distributed cracking and maintain individual crack openings within a healable range under PE-fiber bridging. From the chemical perspective, the availability and transport of reactive species govern the rate and extent of precipitation within these cracks. The proposed crack-width-guided strategy therefore provides a basis for integrating strain-hardening design with intrinsic durability enhancement.

Several limitations remain. The approximate healing boundaries were inferred from a limited number of representative crack widths, and quantitative crack-closure kinetics were not determined. In addition, pH and ionic concentrations were not monitored during wet–dry cycling, and only one FAC replacement level was investigated. Future studies should combine controlled crack-width generation, time-resolved three-dimensional crack characterization, chemical monitoring of the healing environment, and systematic FAC-content optimization to establish quantitative links among crack geometry, reaction kinetics, and transport-property recovery.

## Figures and Tables

**Figure 1:**
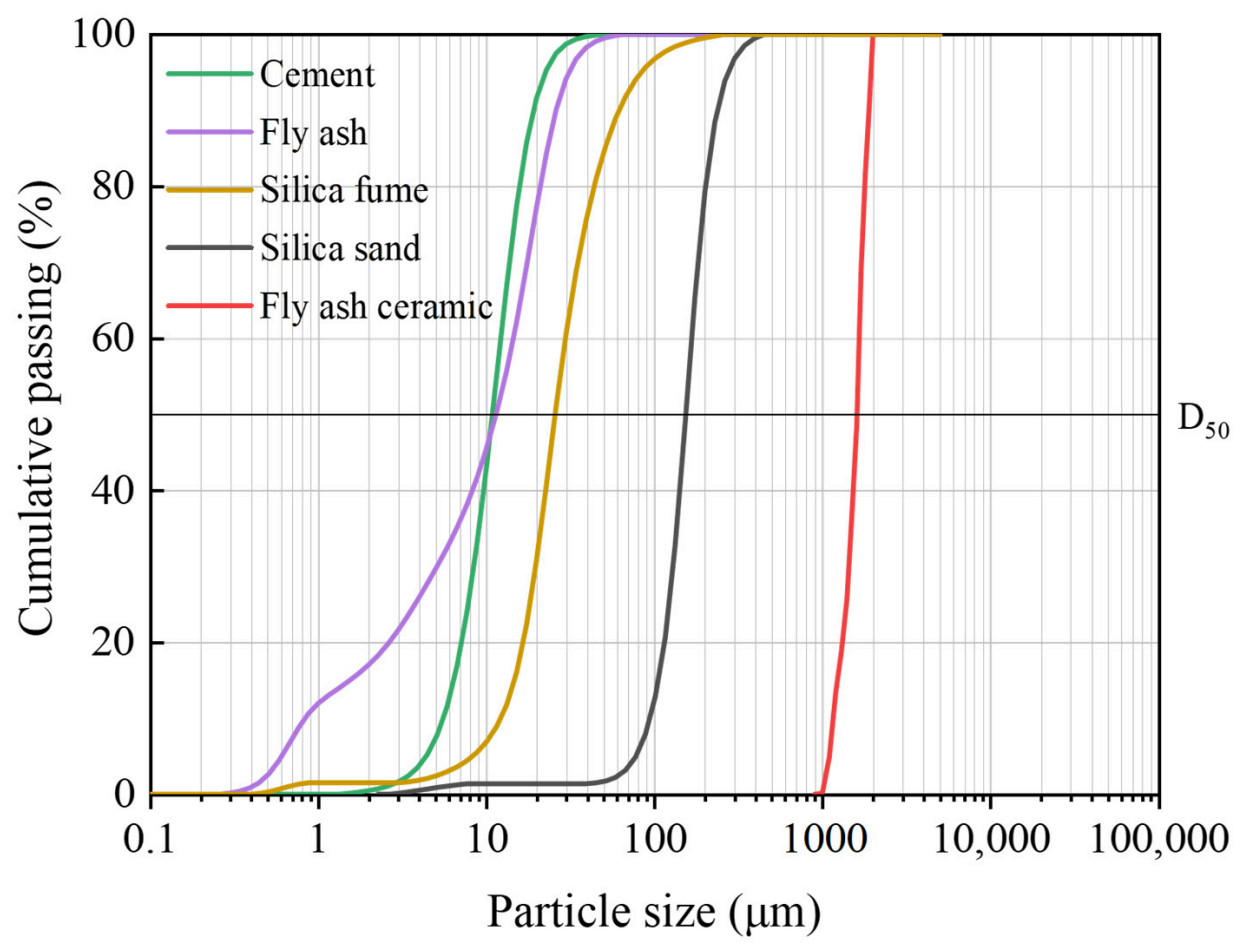
Particle size distribution of solid ingredients.

**Figure 2:**
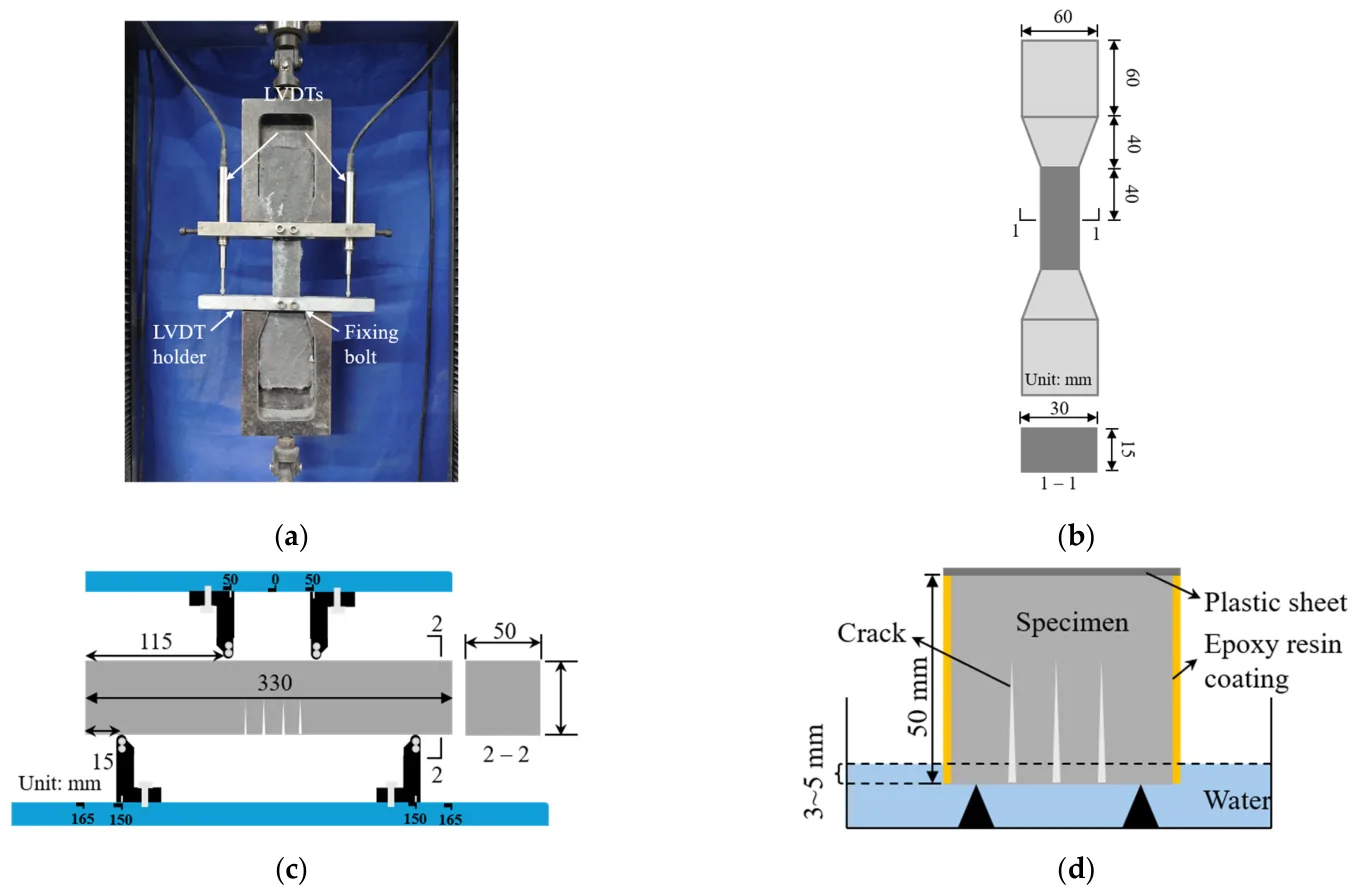
Schematic for (**a**) uniaxial tensile test setup, (**b**) dumbbell specimen, (**c**) pre-cracking of beam under four point bending test, and (**d**) water sorptivity test.

**Figure 3:**
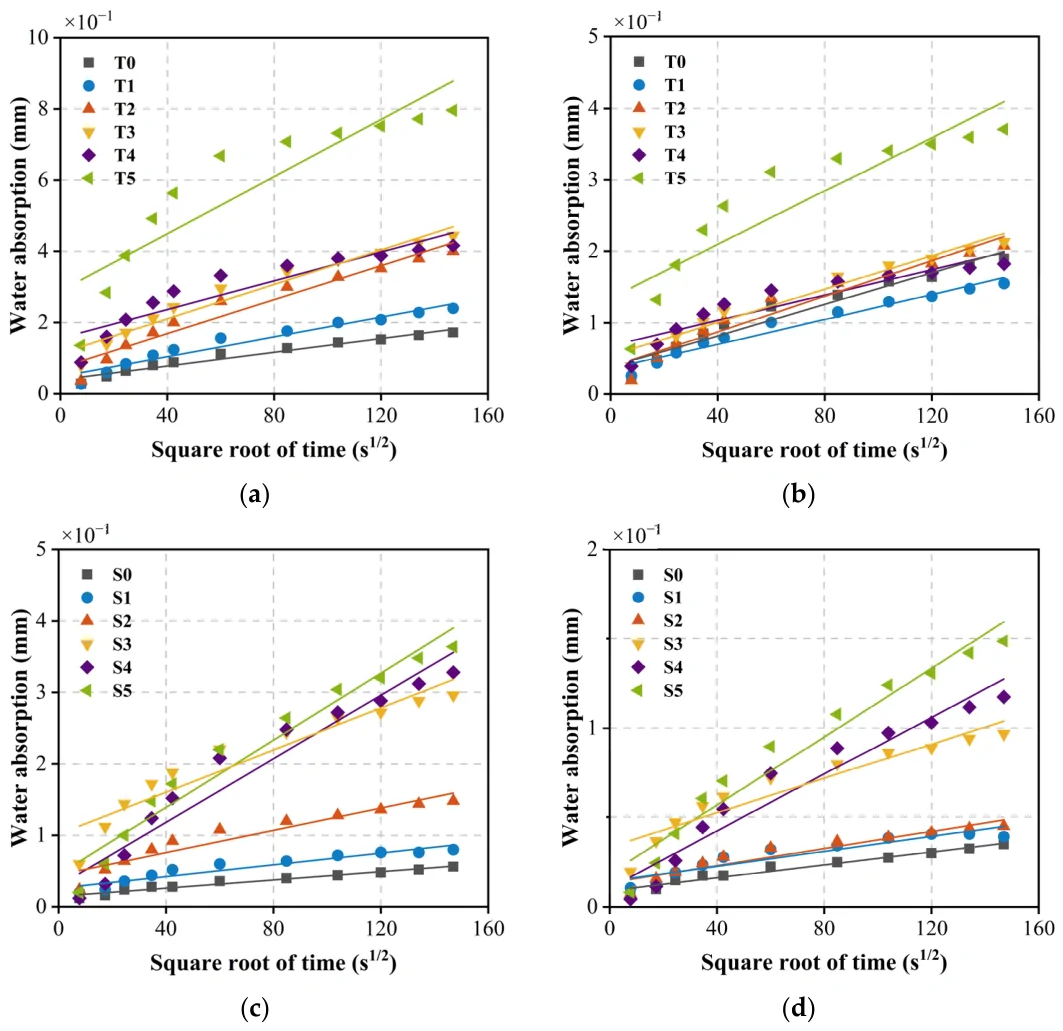
Relationship between water absorption and square root of time: (**a**) Before self-healing curing under tap water. (**b**) After self-healing curing under tap water. (**c**) Before self-healing curing under seawater. (**d**) After self-healing curing under seawater.

**Figure 4:**
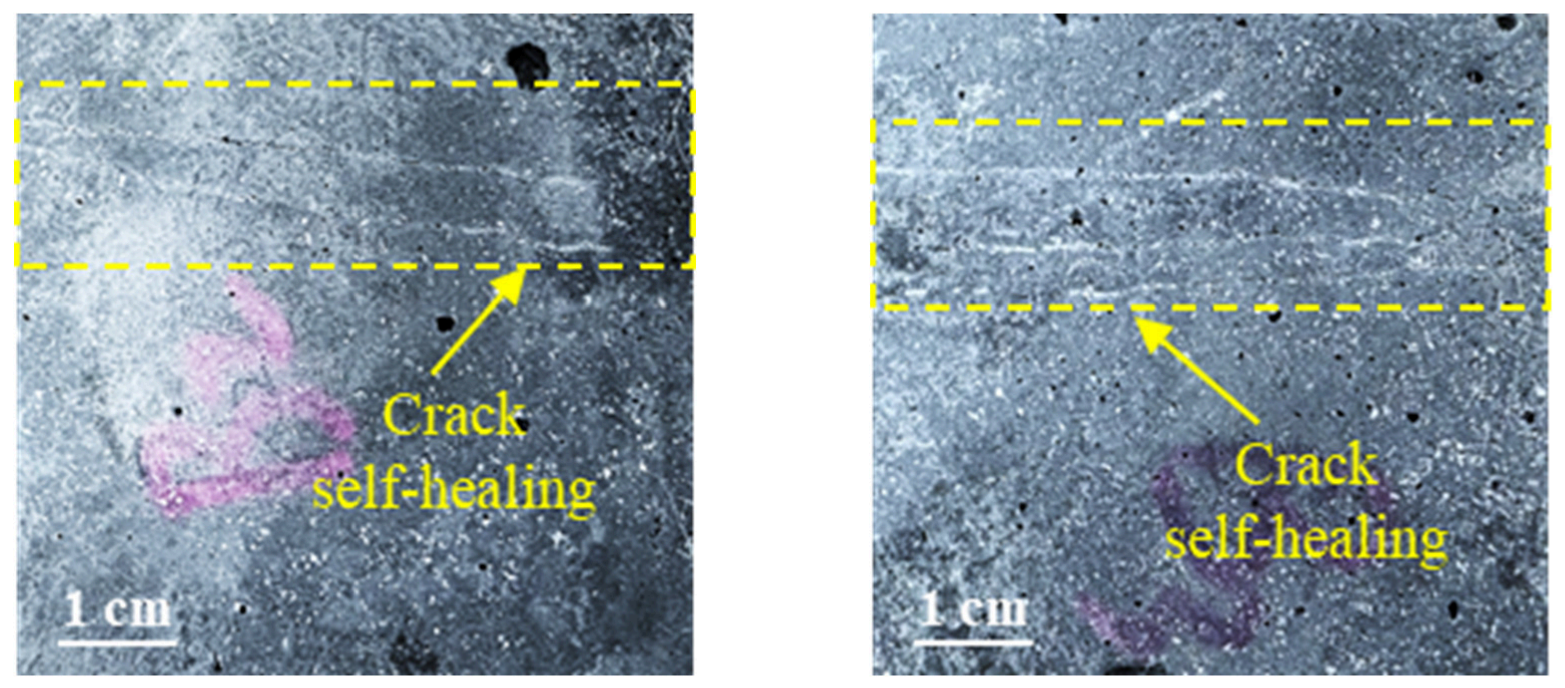
Cracks healed by self-healing products.

**Figure 5:**
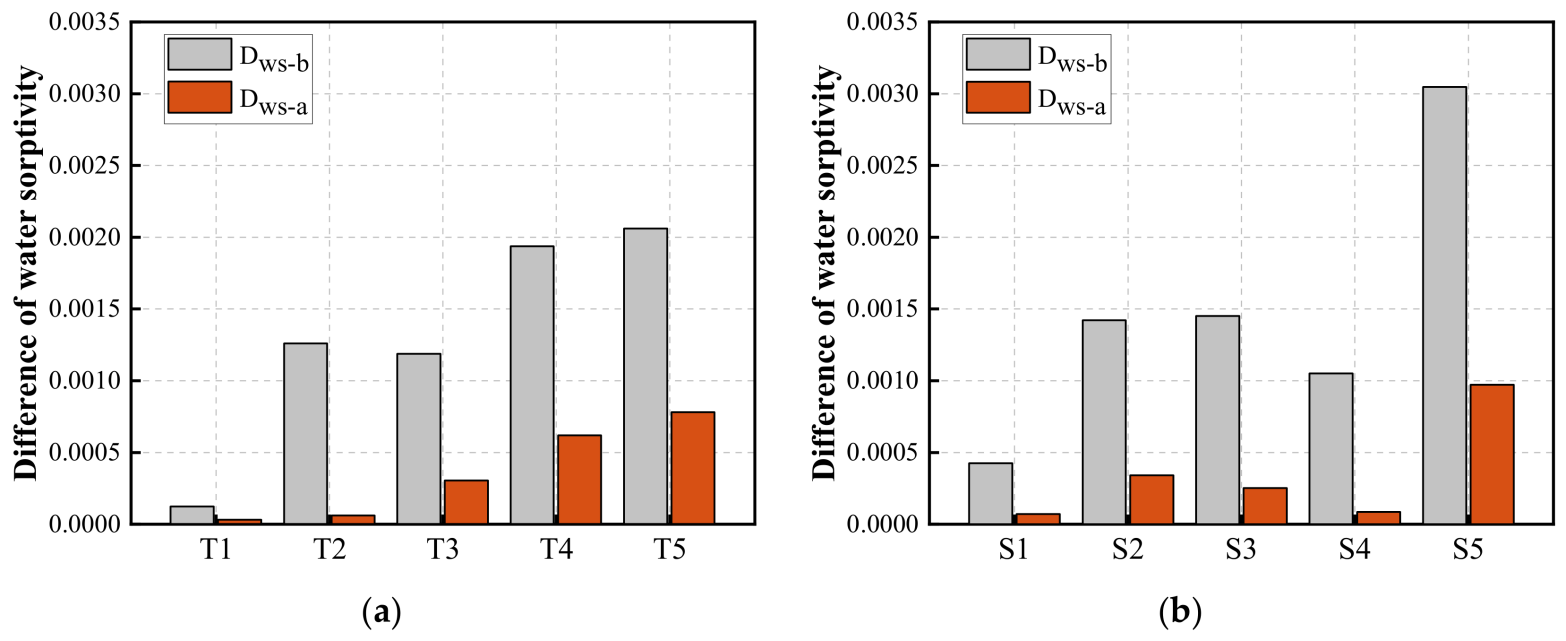
Difference of water sorptivity in different environments: (**a**) Tap water. (**b**) Seawater.

**Figure 6:**
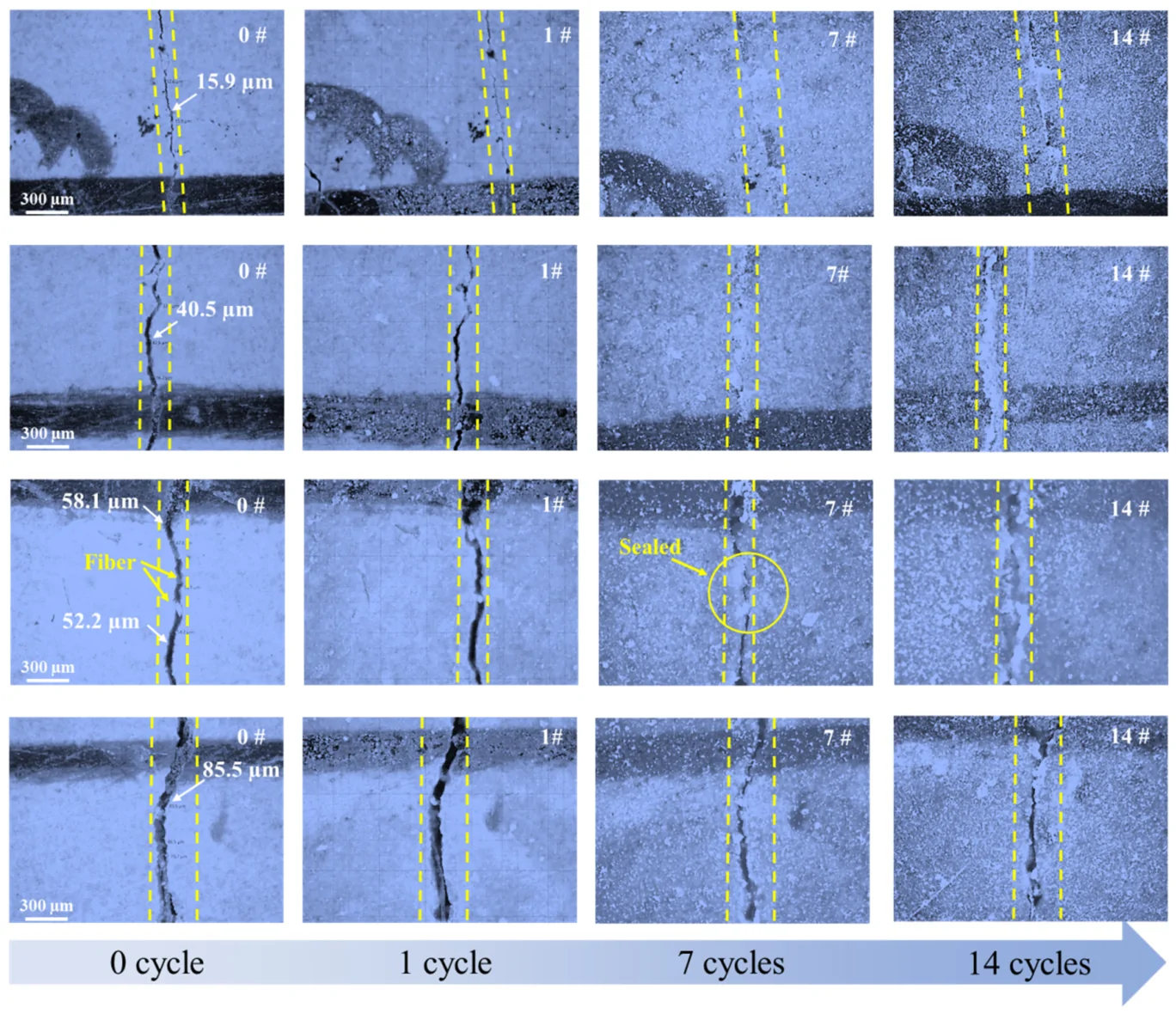
Crack self-healing under tap water as observed via optical microscope.

**Figure 7:**
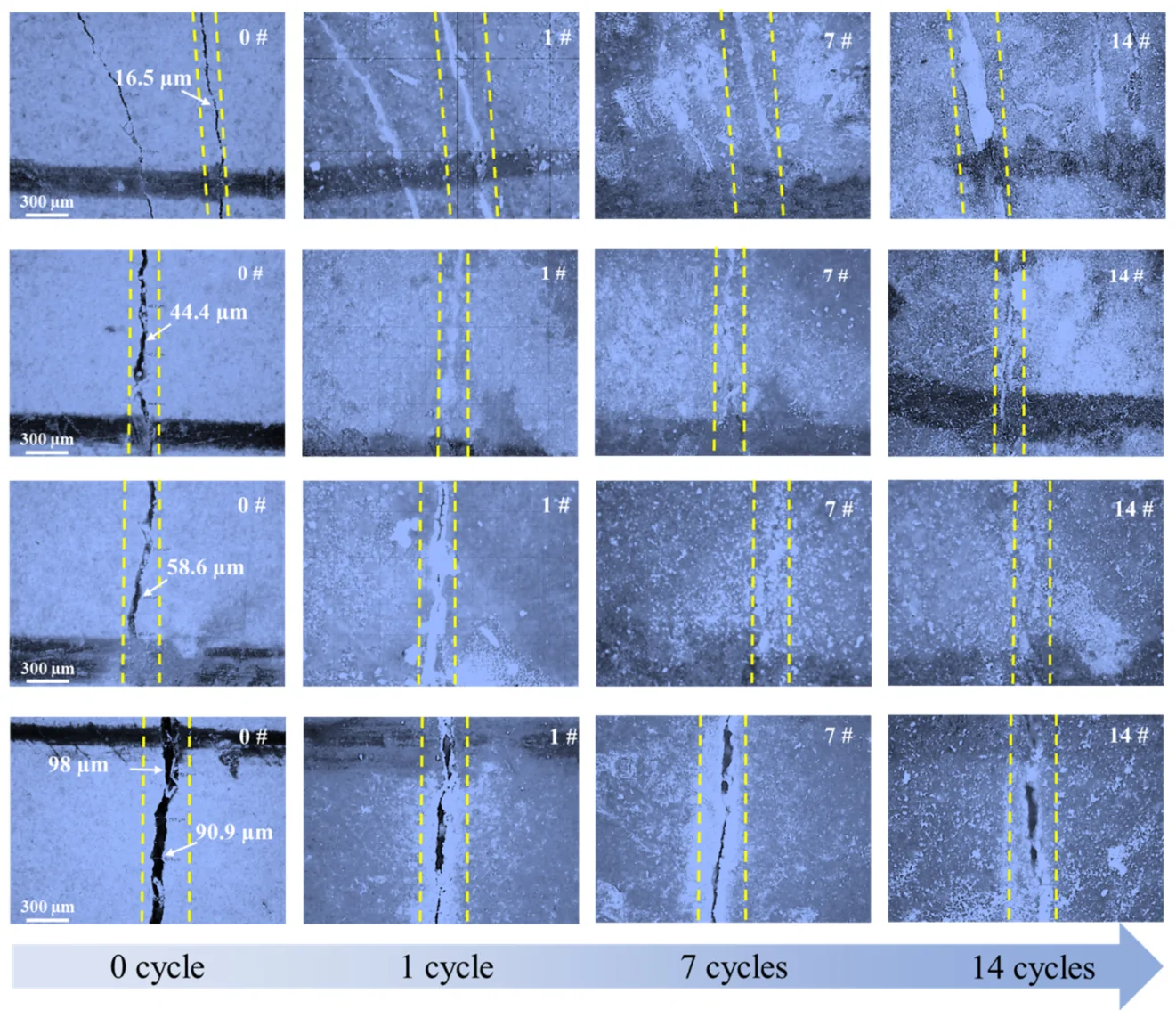
Crack self-healing under seawater wet–dry cycle as observed via optical microscope.

**Figure 8:**
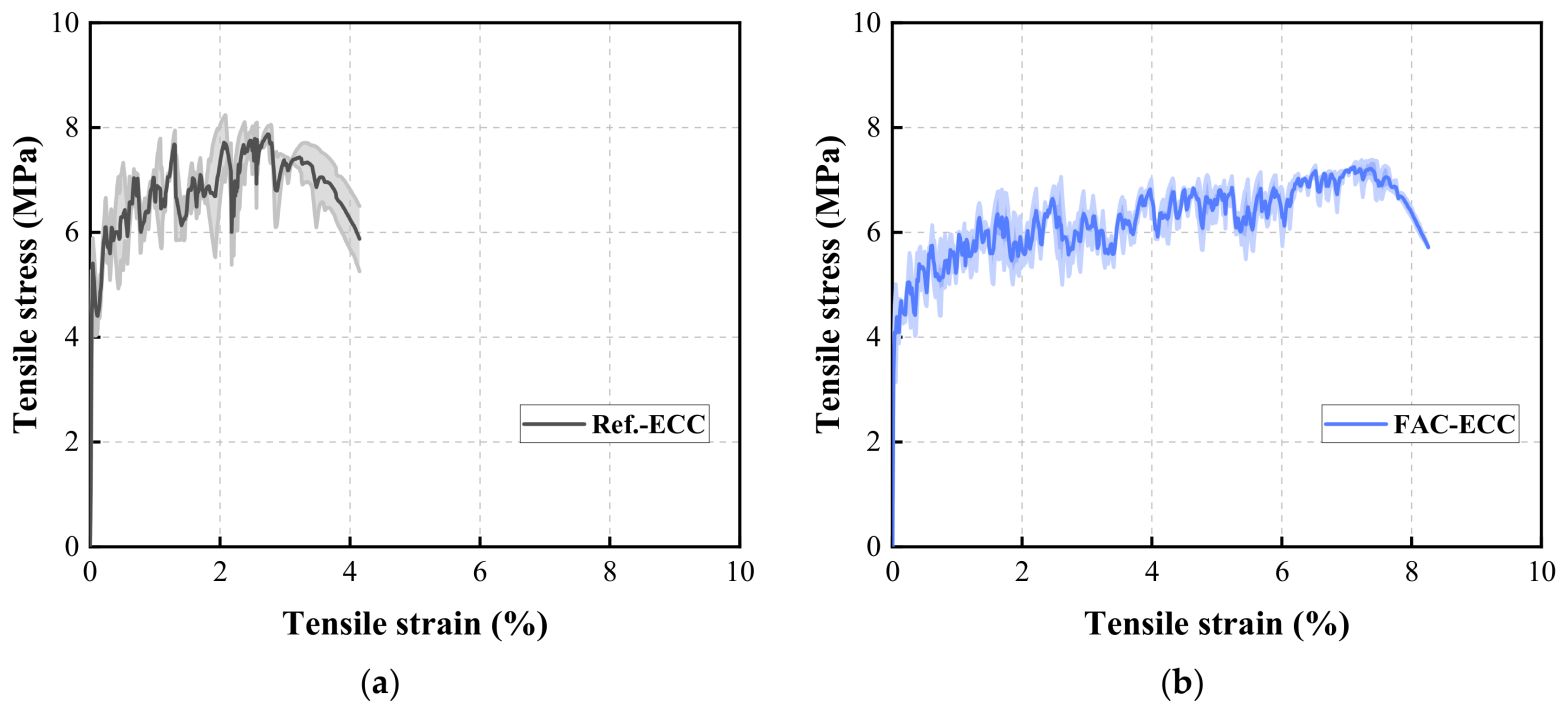
Tensile stress–strain curves of ECC mixtures: (**a**) Ref.-ECC. (**b**) FAC-ECC.

**Figure 9:**
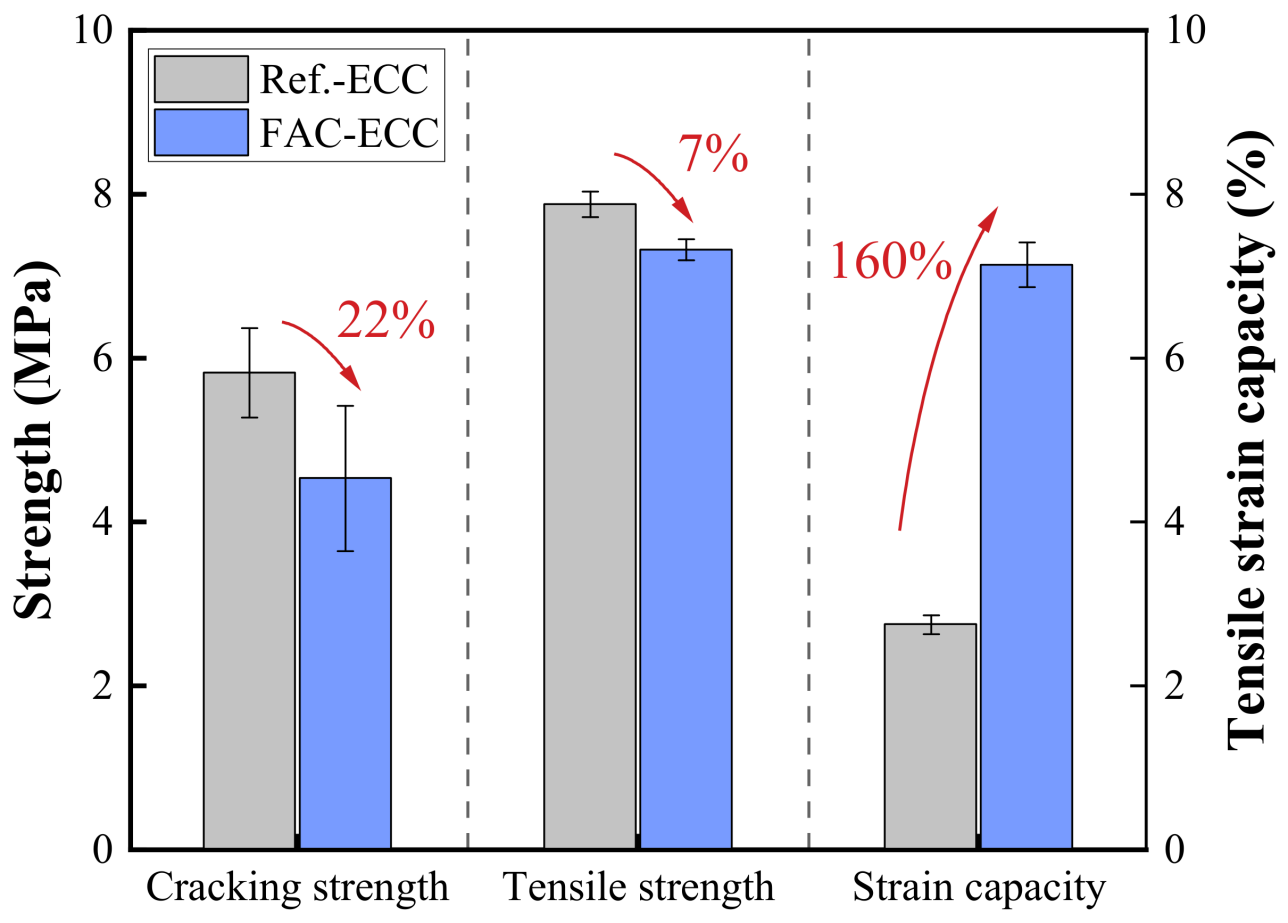
Tensile properties of ECC mixtures. Values are presented as mean ± standard deviation based on three independent specimens (*n* = 3).

**Figure 10:**
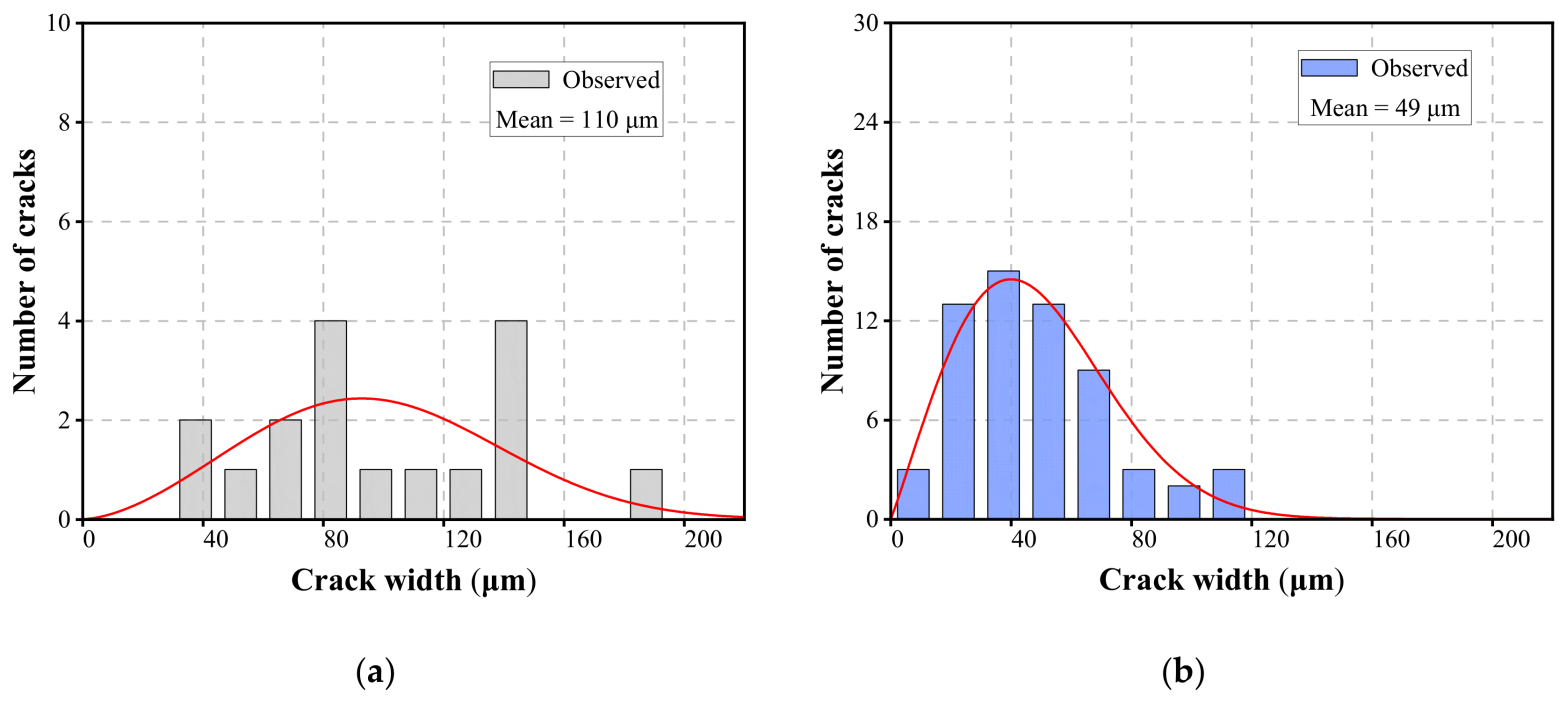
Representative post-test crack-width distributions: (**a**) Ref.-ECC. (**b**) FAC-ECC.

**Figure 11:**
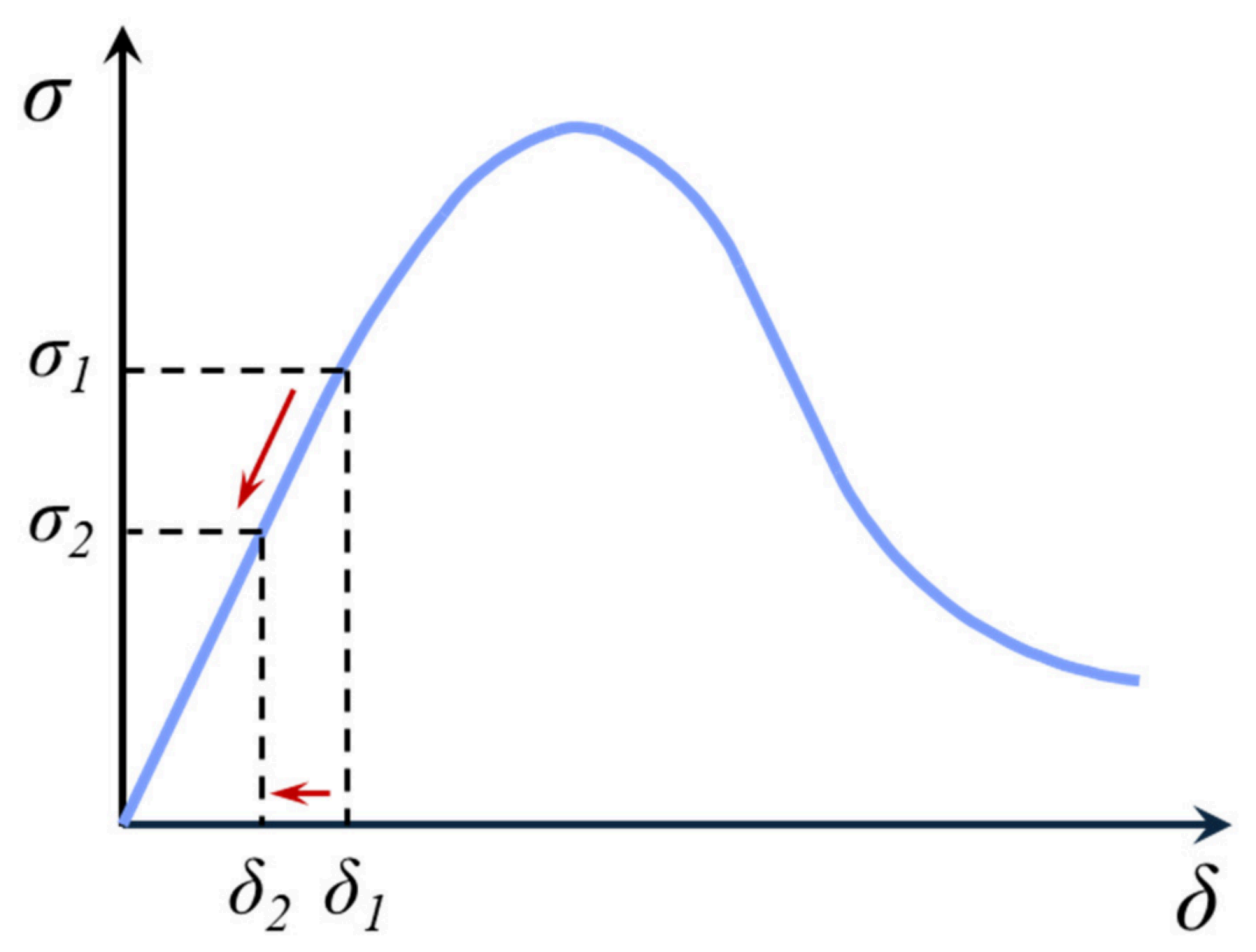
Schematic micromechanical interpretation of the effect of FAC on crack opening.

**Figure 12:**
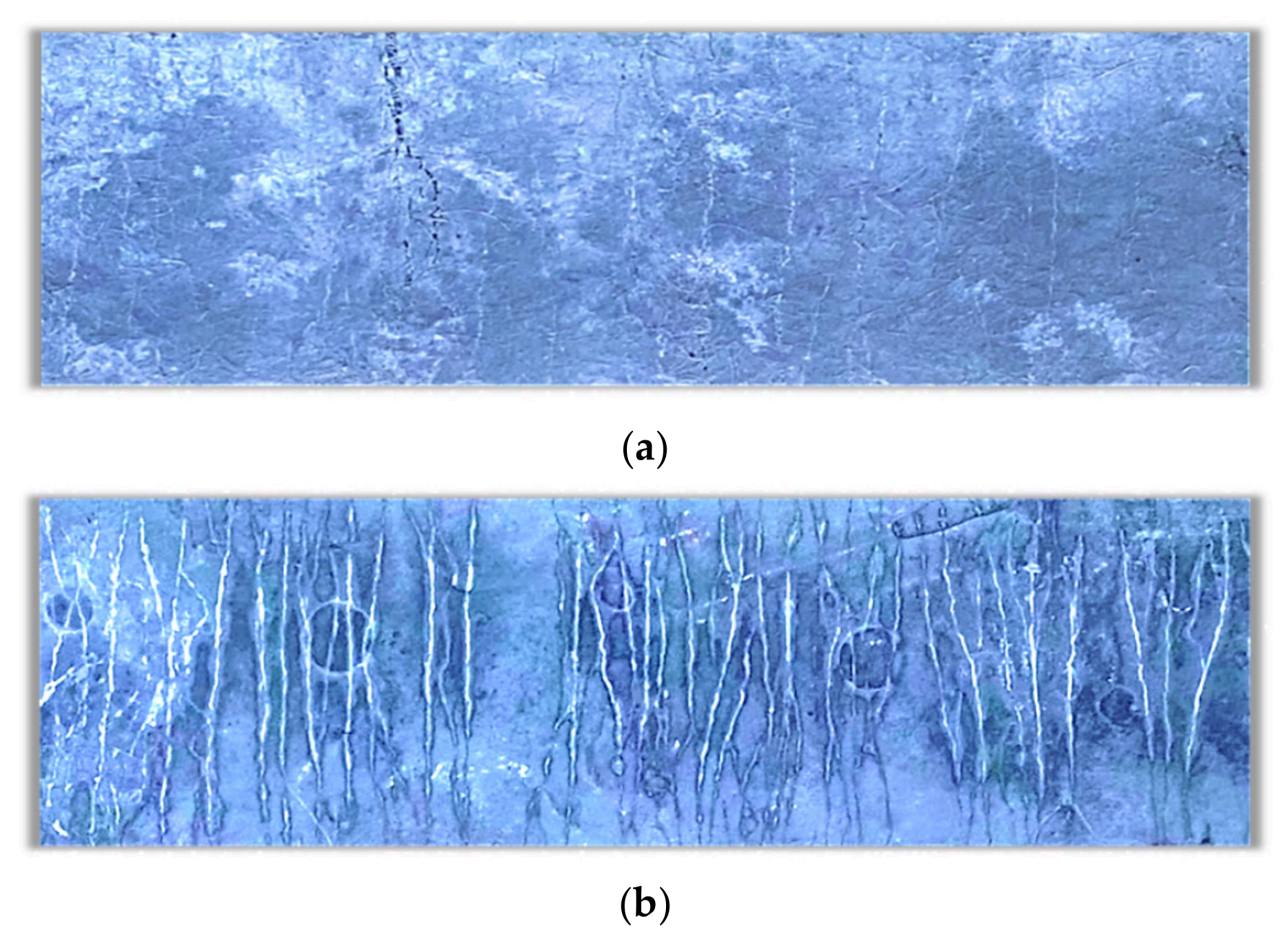
Self-healing behavior in different environments: (**a**) Tap water and (**b**) seawater.

**Figure 13:**
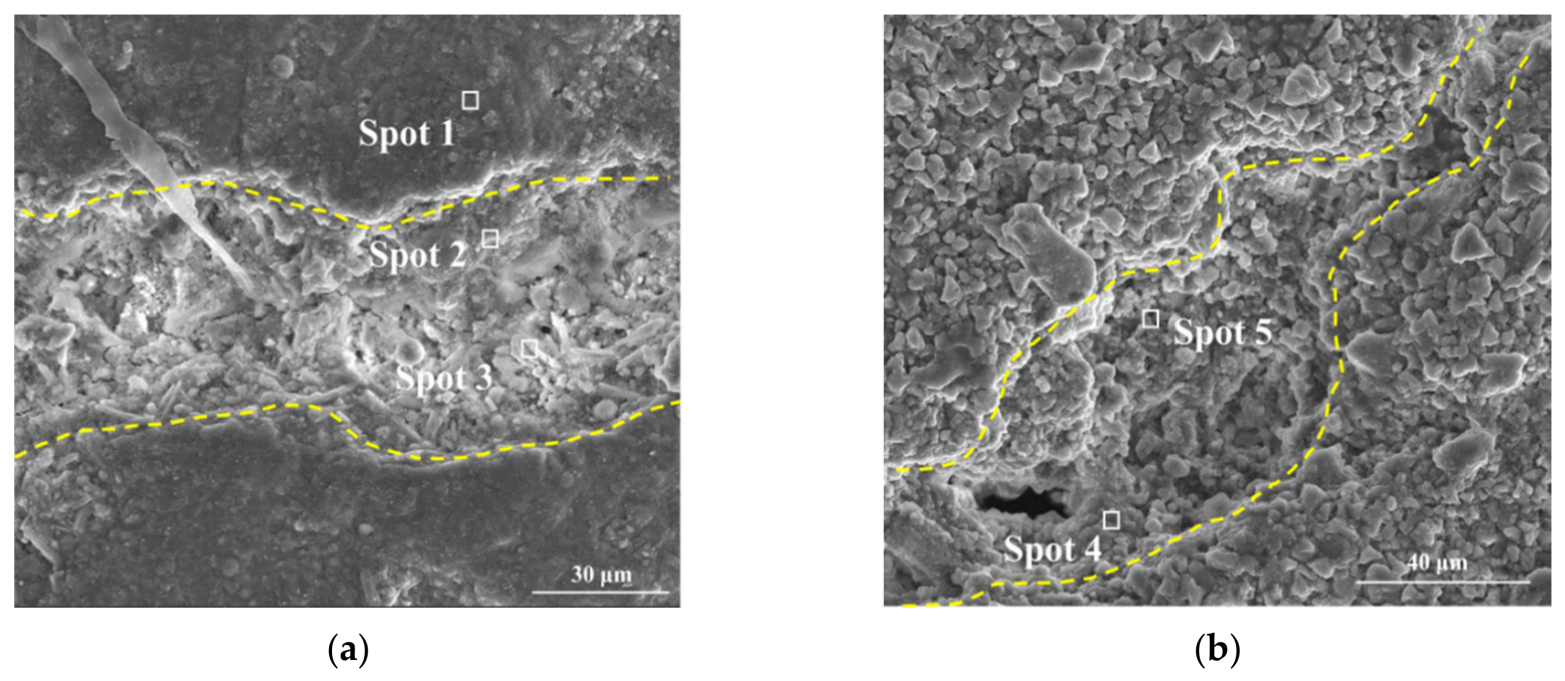
SEM images of self-healing products within crack under different environments: (**a**) Tap water. (**b**) Seawater.

**Figure 14:**
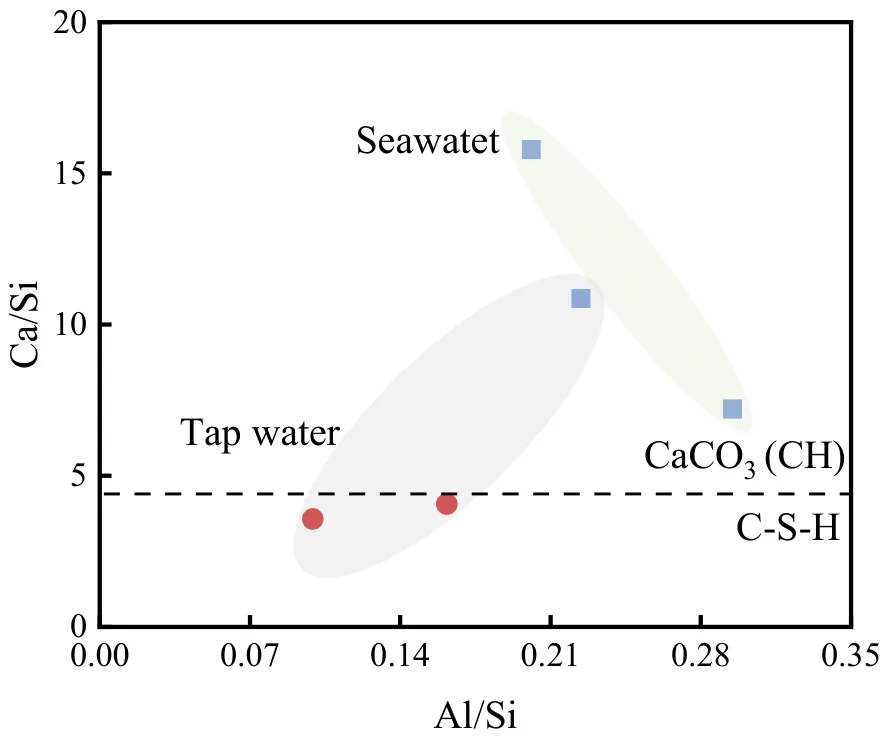
Ca/Si against Al/Si atom ration plot for EDS.

**Figure 15:**
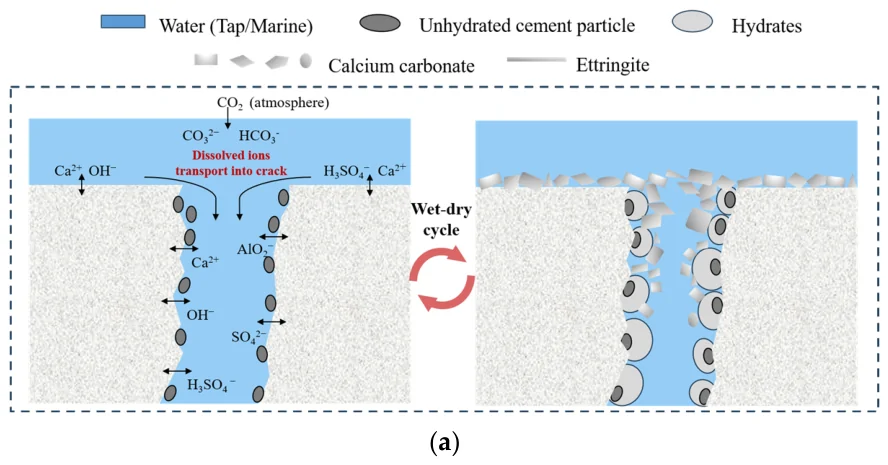

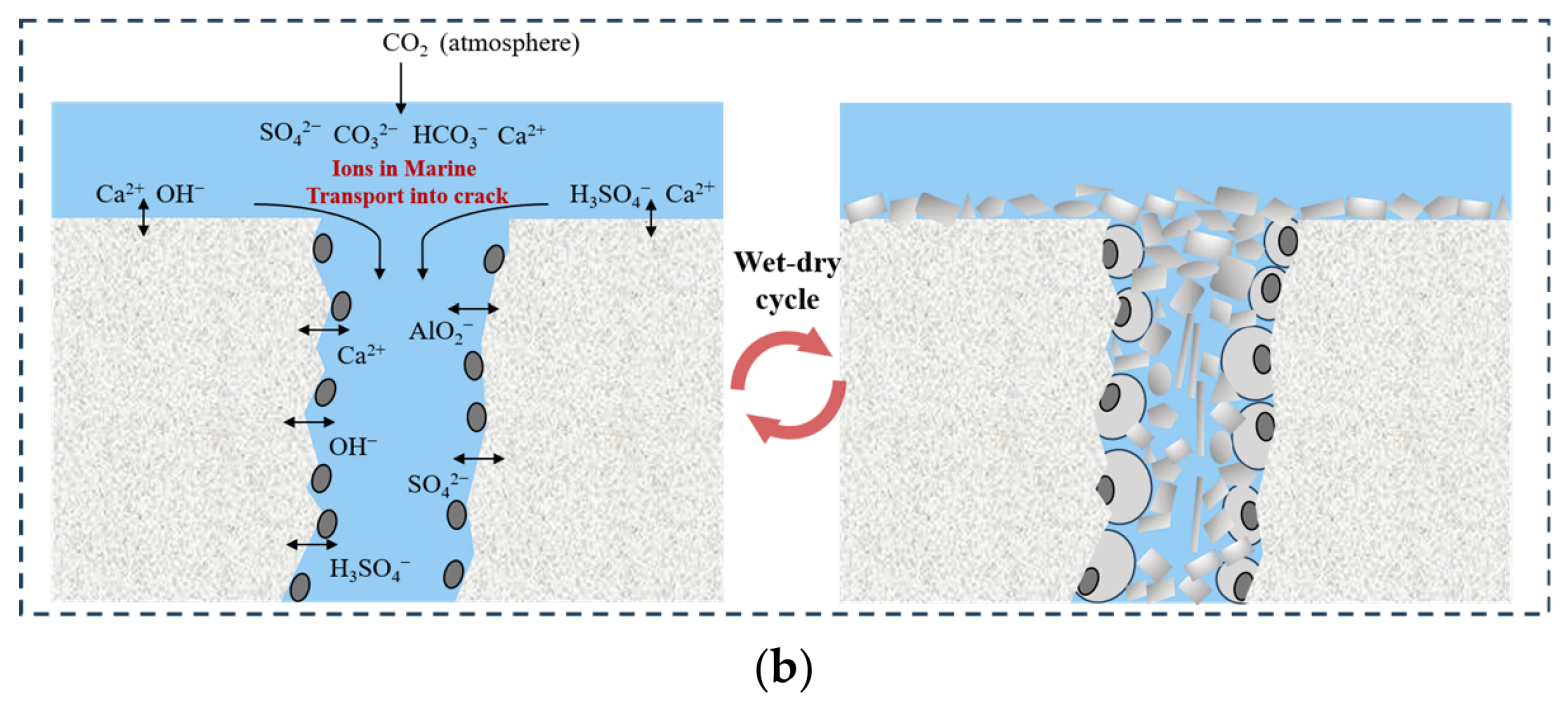
Self-healing mechanism in different environments: (**a**) Cured under tap water. (**b**) Cured under seawater.

**Table 1:** Chemical composition of cement, fly ash, and silica fume (%).

Eliment Type	CaO	SiO_2_	TiO_2_	MgO	Al_2_O_3_	Fe_2_O_3_	Na_2_O	K_2_O	Others
Cement	64.3	23.4	0.6	3.3	3.6	3.8	0.3	0.4	0.3
Silica fume	1.2	95.4	-	0.6	0.5	0.5	0.75	0.8	0.25
Fly ash	28.4	37.8	0.1	6	18.3	7.52	0.3	0.4	1.18

**Table 2:** Mix proportions of ECC mixture (kg/m^3^).

ID	C	FA	SF	W	SS	FAC	SP	PE Fiber
Ref.-ECC	718	575	115	338	442	0	18	19
FAC-ECC	718	575	115	338	282	36	18	19

**Table 3:** Chemical composition of substitute seawater (g/L).

Composition	NaCl	MgCl_2_	Na_2_SO_4_	CaCl_2_	KCl	NaHCO_3_	KBr	H_3_BO_3_	SrCl_3_	NaF
	24.53	5.2	4.09	1.16	0.695	0.201	0.101	0.027	0.025	0.003

**Table 4:** Water sorptivity index (Ws) in different curing environments.

ID	Before Self-Healing		After Self-Healing	
	Tap Water	Seawater	Tap Water	Seawater
T0/S0	2.87 × 10^−4^	9.66 × 10^−4^	1.78 × 10^−4^	7.97 × 10^−4^
T1/S1	4.09 × 10^−4^	13.91 × 10^−4^	2.07 × 10^−4^	8.69 × 10^−4^
T2/S2	15.46 × 10^−4^	23.88 × 10^−4^	2.37 × 10^−4^	12.39 × 10^−4^
T3/S3	14.73 × 10^−4^	24.17 × 10^−4^	4.81 × 10^−4^	11.5 × 10^−4^
T4/S4	22.22 × 10^−4^	20.18 × 10^−4^	7.95 × 10^−4^	8.84 × 10^−4^
T5/S5	23.47 × 10^−4^	40.13 × 10^−4^	9.57 × 10^−4^	18.70 × 10^−4^

**Table 5:** Crack characteristics of representative post-test ECC specimens.

	Ref.-ECC	FAC-ECC
Number of cracks	17	61
Average crack width	110 μm	49 μm

**Table 6:** Chemical composition of self-healing products (%).

	Si	Al	Ca	O	C
Spot-1	9.64	1.56	39.02	37.37	12.41
Spot-2	12.58	1.25	44.69	34.59	6.89
Spot-3	3.3	0.74	35.84	46.27	13.85
Spot-4	6.31	1.86	45.46	39.18	7.19
Spot-5	1.89	0.38	29.83	50.71	17.19

## Data Availability

The original contributions presented in this study are included in the article. Further inquiries can be directed to the corresponding author.
